# Supplementary material for: The Required Competencies of Bachelor- and Master-Educated Nurses in Facilitating the Development of an Effective Workplace Culture in Nursing Homes: An Integrative Review
Source: Int J Environ Res Public Health. 2022 Sep 28;19(19):12324. doi: 10.3390/ijerph191912324 (PMC9564543; doi:10.3390/ijerph191912324)
Supplement: Supplementary file 1 [file ijerph-19-12324-s001.zip › ijerph-1882415-supplementary.pdf]

Supplementary table S1

Table S1. The search strategy in databases.

	English Keywords<br>PubMED		English Keywords<br>CINAHL		English Keywords<br>PsycINFO
**#1: Target group**	Bachelor[Title/Abstract]<br>Baccalaureate<br>Bachelor's<br>Undergraduate<br>BSN<br>practitioner *<br>Master *<br>scientist *<br>science<br>MANP<br>advanced practice<br>advanced nursing practice<br>specialist *<br>Undergraduate<br>Research-based<br>Theory-based<br><br>**AND**<br>Nurse<br>Nurses<br>Nursing	Education, Nursing, Baccalaureate [Mesh]<br>Nursing Education Research [Mesh]<br>Education, Nursing, Graduate [Mesh]<br>Education, Nursing, Continuing [Mesh]<br>Nurses [Mesh]<br>Nurse's Role [Mesh]	**Ti**<br>BSN<br>Advanced nursing practice<br>MANP<br>Advanced practice<br>Baccalaureate<br>Bachelor<br>Bachelor's<br>Master<br>Practitioner<br>Science<br>Scientist<br>Specialist *<br>Undergraduate<br>Researched-based<br>Theory-based<br><br>**AB**<br>BSN<br>Advanced nursing practice<br>MANP<br>Advanced practice<br>Baccalaureate<br>Bachelor<br>Bachelor's<br>Master	MH Education, Nursing, Research-Based<br>MH Education, Nursing, Theory-Based<br>MH Baccalaureate Nurses<br>MH Education, Nursing, Baccalaureate+<br>MH Education, Nursing, Graduate+<br>MH Advanced Nursing Practice<br>MH Advanced Practice Nurses<br>MH Gerontological Advanced Practice Nurses Association<br>MH Masters-Prepared Nurses<br>MH Nursing Practice, Evidence-Based<br>MH Education, Nursing, Masters<br>MH Nursing Researchers<br><br>**AND**<br>MH Nurses+<br>MH Nursing role<br>MH Nurses by role+<br>MH Nursing Leaders<br>MH Nursing Practice+	**Ti.ab.**<br>Evidence based practice<br>BSN<br>MANP<br>Advanced nursing practice<br>Advanced practice<br>Baccalaureate<br>Bachelor<br>Bachelor's<br>Master *<br>Practitioner *<br>Science<br>Scientist<br>Specialist *<br>Undergraduate<br>Nurses<br>Nursing education<br>Nursing<br>Nurse<br>Nurses

			Practitioner<br>Science<br>Scientist<br>Specialist *<br>Undergraduate<br>Researched-based<br>Theory-based	MH Gerontologic Nurse Practitioners<br>MH Education, Nursing, Continuing<br>MH Nursing Homes+<br>MH Housing for the elderly<br>MH Long Term Care<br>MH Nursing Home Patients<br>MH Residential care		
**#2: Work context**	Nursing home *<br>Long term care<br>Long term healthcare<br>Long term health care<br>Elderly care<br>Gerontological nurs *<br>Geriatric nurs *<br>Residential care<br>Residential facilit *<br>Housing for the elderly<br>Retirement home *	Nursing home * [Mesh]<br>Residential Facilities [Mesh]<br>Long-Term Care [Mesh]	**Ti en AB**<br>Nursing homes<br>Long term care<br>Elderly care<br>Gerontological nursing<br>Residential facility<br>Residential care<br>Housing for the elderly		Geriatric nurs *<br>Gerontological nurs *<br>Housing for the elderly<br>Long term health care<br>Nursing home *<br>Residential care<br>Residential facility *<br>Retirement home *	Exp Nursing homes<br>Residential care institutions<br>Long term care<br>Elder care
**#3: Competencies**	Competencies<br>Competenc *<br>Clinical competenc *<br>Nurse competenc *<br>Professional competence<br><br>**<u>Translation synonyms</u>**<br>Abilit *<br>Expertise	Professional competence [Mesh]<br>Clinical competence [Mesh]<br>Academic success [Mesh]<br>Attitude [Mesh]	**Ti en AB**<br>Competencies<br>Competetenc *<br>Clinical competenc *<br>Nurse competenc *<br>Professional competence<br>Ability *	MH Professional Competence+<br>MH Academic Performance<br>MH Attitude<br>MH Attitude of Health Personnel<br>MH Nurse Attitudes<br>MH Attitude to Health<br>MH Cultural sensitivity	Competencies<br>Competence *<br>Clinical competenc *<br>Nurse competenc *<br>Professional competence<br>Abilit *<br>Expertise	Exp Professional competence<br>Exp Performance<br>Exp attitudes<br>Health personnel attitudes<br>Attitudes<br>Cultural sensitivity<br>Cultural change

	Capability	Health Knowledge,	Expertise	MH Personal Satisfaction	Capability	Exp employee
	Skill *	Attitudes, Practice [Mesh]	Capability	MH Job satisfaction	Skill *	attitudes
	Skills mix *	Social Skills [Mesh]	Skill *	MH Social attitudes	Skills mix *	Exp job satisfaction
	Skill mix *	Metacognition [Mesh]	Skills mix *	MH Health knowledge	Skill mix *	Social behavior
	Skillmix *	Social Networking [Mesh]	Skill mix *	MH Professional knowledge+	Skillmix *	Leadership
	Tasks	Communication [Mesh]	Skillmix *	MH Social Skills	Tasks	Leadership style
	Role *	Mentoring [Mesh]	Tasks	MH Skill Mix+	Role	Organizational
	Knowledge	Self-concept [Mesh]	Role *	MH Personnel Staffing and	Knowledge	behavior
	Professional attitude		Knowledge	Scheduling	Professional	Responsibility
	Attitude	Emotional Intelligence	Professional	MH Work Assignements+	attitude	Social acceptance
	Behaviour	[Mesh]	attitude	MH Skill Acquisition	Behavior	Social networks
	Behavior	Creativity [Mesh]	Behaviour	MH Skill Retention	Behaviour	Social perception
	Communicati *	Thinking [Mesh]	Behavior	MH Lifelong learning	Communicati *	Social skills
	Leadership	Leadership [Mesh]	Communicati *	MH Personal Growth	Leadership	Exp health
	Organizational	Communication barriers	Leadership	MH Problem Identification	Organizational	knowledge
	Organisational	[Mesh]	Organizational	MH Problem Solving+	Organisational	Professional
	Analytic *	Nonverbal	Organizational	MH Transfer	Analytic *	development
	Critical thinking	communication [Mesh]	Analytic *	MH Learning+	Critical thinking	Development
	Awareness	Verbal behavior [Mesh]	Critical thinking	MH Cognition	Awareness	Exp social skills
	Creativity	Mentors [Mesh]	Awareness	MH Consciousness	Creativity	Working conditions
	Empathy	Personality development	Creativity	MH Intuition	Empathy	Skill learning
	Networking	[Mesh]	Empathy	MH Communication	Networking	Learning
	Persuasiveness	Personality [Mesh]	Networking	MH Communication Barriers	Persuasiveness	Learning strategies
	Decisiveness	Identity crises [Mesh]	Persuasiveness	MH Communication Skills	Decisiveness	Personality
	Coaching	Individuation [Mesh]	Decisiveness	MH Conversation	Coaching	development
	Flexibility	Intelligence [Mesh]	Coaching	MH Nonverbal	Flexibility	Exp problem based
	Self confidence	Evidence-Based Nursing	Flexibility	communication	Self confidence	learning
	Integrity	[Mesh]	Self confidence	MH Social Networking	Integrity	Exp decision making
	Vision		Integrity	MH Verbal Behavior+	Vision	Problem solving
	Entrepreneur		Vision	MH Body Language	Enerpreneur	Declarative
	Conversation		Entrepreneur	MH Presence	Conversation	knowledge
	Nonverbal		Conversation	MH Mentorship	Nonverbal	Inductive deductive
	communication			MH Personality Development	communication	reasoning

	Body language<br>Presence<br>Mentor *<br>Personality development<br>Self concept<br>Personal boundaries<br>Identity crisis<br>Individuation<br>Intelligence<br>Reflect *<br>Evidence based practice<br>Evidence based nursing	Nonverbal communication<br>Body language<br>Presence<br>Mentor *<br>Personality development<br>Self concept<br>Personal boundaries<br>Identity crisis<br>Individuation<br>Intelligence<br>Reflect *<br>Evidence based practice<br>Evidence based nursing	MH Self concept+<br>MH Identity Crisis<br>MH Individuation<br>MH Professional Boundaries<br>MH Personal Boundaries+<br>MH Intelligence+<br>MH Thinking+<br>MH Personality+<br>MH Reflection	Body language<br>Presence<br>Mentor *<br>Personality development<br>Self concept<br>Personal boundaries<br>Identity crises<br>Individuation<br>Intelligence<br>Reflect *<br>Evidence based practice<br>Evidence based nursing	Reasoning<br>Exp knowledge (general)<br>Cognitive processes<br>Metacognition<br>Exp intuition<br>Communication skills<br>Exp ability<br>Exp conversation<br>Communication<br>Nonverbal communication<br>Persuasive communication<br>Verbal communication<br>Mentor<br>Identity crisis<br>Professional standards<br>Intelligence<br>Emotional intelligence<br>Thinking<br>Critical thinking<br>Logical thinking<br>Rationality<br>Reasoning<br>Personality<br>Self-actualization<br>Self-concept<br>Self-determination

		Self-expression<br>Self-monitoring (personality)<br>Cognitive style<br>Reflectiveness<br>feedback

*Search for word variations and words that begin with a word stem

	**PubMed**	
		Results
#1 Target group	("Nursing Education Research"[Mesh] OR "Education, Nursing, Baccalaureate"[Mesh] OR "Education, Nursing, Graduate"[Mesh] OR BSN[tiab] OR MANP[tiab] OR advanced nursing practice[tiab] OR ((advanced practice [tiab] OR Baccalaureate[tiab] OR Bachelor [tiab] OR Bachelor's [tiab] OR Master*[tiab] OR practitioner*[tiab] OR science[tiab] OR scientist*[tiab] OR specialist*[tiab] OR Undergraduate[tiab] OR researched-based[tiab] OR theory-based[tiab]) AND (Nurses [Mesh] OR "Nurse's Role"[Mesh] OR "Education, Nursing, Continuing"[Mesh] OR nurse[tiab] OR Nurses[tiab] OR Nursing[tiab])))	**#1: 79.553**
#2 Work context	(Nursing home [Mesh] OR Residential Facilities [Mesh] OR "Long-Term Care"[Mesh] OR Elderly care[tiab] OR Geriatric nurs*[tiab] OR Gerontological nurs*[tiab] OR Housing for the elderly[tiab] OR Long term care[tiab] OR Long term health care[tiab] OR Long term healthcare[tiab] OR Nursing home*[tiab] OR Residential care[tiab] OR Residential facilit*[tiab] OR Retirement home*[tiab])	**#2: 97.025**
#3 Competencies	"professional competence"[MeSH Terms] OR "clinical competence"[MeSH Terms] OR "academic success"[MeSH Terms] OR "attitude"[MeSH Terms] OR "health knowledge, attitudes, practice"[MeSH Terms] OR "social skills"[MeSH Terms] OR "metacognition"[MeSH Terms] OR "social networking"[MeSH Terms] OR "communication"[MeSH Terms] OR "mentoring"[MeSH Terms] OR "self concept"[MeSH Terms] OR "emotional intelligence"[MeSH Terms] OR "creativity"[MeSH Terms] OR "thinking"[MeSH Terms] OR "Leadership"[MeSH Terms] OR "communication barriers"[Mesh Terms] OR "nonverbal communication"[Mesh Terms] OR "verbal behavior"[Mesh Terms] OR "mentors"[MeSH Terms] OR "personality development"[Mesh Terms] OR "identity crisis"[Mesh terms] OR "individuation"[Mesh Terms] OR "intelligence"[Mesh Terms] OR "personality" [Mesh Terms] OR "Evidence-Based Nursing"[MeSH Terms]<br>OR	**#3: 8,481,999**

	"Competencies"[Title/Abstract] OR "competenc*"[Title/Abstract] OR "clinical competenc*"[Title/Abstract] OR "nurse competenc*"[Title/Abstract] OR "professional competence"[Title/Abstract] OR "abilit*"[Title/Abstract] OR "Expertise"[Title/Abstract] OR "Capability"[Title/Abstract] OR "skill*"[Title/Abstract] OR "skills mix*"[Title/Abstract] OR "skill mix*"[Title/Abstract] OR "skillmix*"[Title/Abstract] OR "Tasks"[Title/Abstract] OR "role*"[Title/Abstract] OR "Knowledge"[Title/Abstract] OR "professional attitude"[Title/Abstract] OR "Behaviour"[Title/Abstract] OR "Behavior"[Title/Abstract] OR "communicati*"[Title/Abstract] OR "Leadership"[Title/Abstract] OR "organizational"[Title/Abstract] OR "organisational"[Title/Abstract] OR "analytic*"[Title/Abstract] OR "critical thinking"[Title/Abstract] OR "awareness"[Title/Abstract] OR "creativity"[Title/Abstract] OR "empathy"[Title/Abstract] OR "networking"[Title/Abstract] OR "persuasiveness"[Title/Abstract] OR "decisiveness"[Title/Abstract] OR "coaching"[Title/Abstract] OR "flexibility"[Title/Abstract] OR "self confidence"[Title/Abstract] OR "integrity"[Title/Abstract] OR "vision"[Title/Abstract] OR "enterpreneur*"[Title/Abstract] OR "conversation" OR "nonverbal communication" OR "body language" OR "presence" OR "mentor*"[Title/Abstract] OR "personality development"[Title/Abstract] OR "self concept" OR "personal boundaries" OR "identity crisis"[Title/Abstract] OR "individuation"[Title/Abstract] OR "intelligence"[Title/Abstract] OR "reflect*" [Title/Abstract] OR "evidence based practice"[Title/Abstract] OR "evidence based nursing"[Title/Abstract]	
	**#1 AND #2 AND #3**	**2.092**
	**Filter by publication date <10 year**	**1.075**
	**CINAHL**	
#1 Target group	(MH "Education, Nursing, Research-Based") OR (MH "Research, Nursing") OR (MH "Education, Nursing, Masters") OR (MH "Education, Nursing, Theory-Based") OR (MH "Baccalaureate Nurses") OR (MH "Education, Nursing, Baccalaureate+") OR (MH "Education, Nursing, Graduate+") OR (MH "Advanced Nursing Practice+") OR (MH "Advanced Practice Nurses+") OR (MH "Gerontological Advanced Practice Nurses Association") OR (MH "Masters-Prepared Nurses") OR (MH "Nursing Practice, Evidence-Based+") OR (MH "Education, Nursing, Masters") OR (MH "Nurse Researchers") OR (MH "Advanced Practice Nurses+") OR (MH "Nurse Researchers") OR TI ("bsn" OR "advanced nursing practice" OR "MANP") OR AB ("bsn" OR "advanced nursing practice" OR "MANP") OR ((TI ("advanced practice" OR "baccalaureate" OR "bachelor" OR **"Bachelor's"** OR **Master*** OR "practitioner*" OR "science" OR "scientist*" OR "specialist*" OR "undergraduate" OR "researched-based" OR "theory-based") OR AB ("advanced practice" OR "baccalaureate" OR "bachelor" OR **"Bachelor's"** OR **Master*** OR "practitioner*" OR "science" OR "scientist*" OR "specialist*" OR "undergraduate" OR "researched-based" OR "theory-based")) AND ((MH "Nurses+") OR (MH "Nursing Role") OR (MH "Nurses by Role+") OR (MH "Nursing Leaders") OR (MH "Nursing Practice+") OR	**#1: 139.909**

	(MH "Gerontologic Nurse Practitioners") OR (MH "Education, Nursing, Continuing") OR TI ("nurse" OR "nurses" OR "nursing") OR AB ("nurse" OR "nurses" OR "nursing")))	
#2 *Work context*	(MH "Nursing Homes+") OR (MH "Housing for the Elderly") OR (MH "Long Term Care") OR (MH "Nursing Home Patients") OR (MH "Residential Facilities") OR (MH "Residential Care") OR TI ("Nursing homes" OR "long term care" OR "elderly care" OR "gerontological nursing" OR "residential facility" OR "residential care" OR "housing for the elderly") OR AB ("Nursing homes" OR "long term care" OR "elderly care" OR "gerontological nursing" OR "residential facility" OR "residential care" OR "housing for the elderly")	**#2: 76.363**
#3 *Competencies*	(MH "Professional Competence+") OR (MH "Academic Performance") OR (MH "Attitude") OR (MH "Attitude of Health Personnel") OR (MH "Nurse Attitudes") OR (MH "Attitude to Health") OR (MH "Cultural Sensitivity") OR (MH "Personal Satisfaction") OR (MH "Job Satisfaction") OR (MH "Social Attitudes") OR (MH "Health Knowledge") OR (MH "Professional Knowledge+") OR (MH "Social Skills") OR (MH "Skill Mix+") OR (MH "Personnel Staffing and Scheduling") OR (MH "Work Assignments+") OR (MH "Skill Acquisition") OR (MH "Skill Retention") OR (MH "Lifelong Learning") OR (MH "Personal Growth") OR (MH "Problem Identification") OR (MH "Problem Solving+") OR (MH "Transfer (Psychology)") OR (MH "Learning+") OR (MH "Cognition") OR (MH "Consciousness") OR (MH "Intuition") OR (MH "Communication") OR (MH "Communication Barriers") OR (MH "Communication Skills") OR (MH "Conversation") OR (MH "Nonverbal Communication") OR (MH "Social Networking+") OR (MH "Verbal Behavior+") OR (MH "Body Language") OR (MH "Presence") OR (MH "Mentorship") OR (MH "Personality Development") OR (MH "Self Concept+") OR (MH "Identity Crisis") OR (MH "Individuation") OR (MH "Professional Boundaries") OR (MH "Personal Boundaries+") OR (MH "Intelligence+") OR (MH "Thinking+") OR (MH "Personality+") OR (MH "Reflection") OR TI ("Competencies" OR "competenc*" OR "clinical competenc*" OR "nurse competenc*" OR "professional competence" OR "abilit*" OR "Expertise" OR "Capability" OR "skill*" OR "skills mix*" OR "skill mix*" OR "skillmix*" OR "Tasks" OR "role*" OR "Knowledge" OR "professional attitude" OR "Behaviour" OR "Behavior" OR "communicati*" OR "Leadership" OR "organizational" OR "organisational" OR "analytic*" OR "critical thinking" OR "awareness" OR "creativity" OR "empathy" OR "networking" OR "persuasiveness" OR "decisiveness" OR "coaching" OR "flexibility" OR "self confidence" OR "integrity" OR "vision" OR "enterpreneur*" OR "conversation" OR "nonverbal communication" OR "body language" OR "presence" OR "mentor*" OR "personality development" OR "self concept" OR "personal boundaries" OR "identity crisis" OR individuation" OR "intelligence" OR "reflect*" OR "evidence based practice" OR "evidence based nursing")	**#3: 1,709,068**

	OR<br>AB (“Competencies” OR “competenc*” OR “clinical competenc*” OR “nurse competenc*” OR “professional competence” OR “abilit*” OR “Expertise” OR “Capability” OR “skill*” OR “skills mix*” OR “skill mix*” OR “skillmix*” OR “Tasks” OR “role*” OR “Knowledge” OR “professional attitude” OR “Behaviour” OR “Behavior” OR “communicati*” OR “Leadership” OR “organizational” OR “organisational” OR “analytic*” OR “critical thinking” OR “awareness” OR “creativity” OR “empathy” OR “networking” OR “persuasiveness” OR “decisiveness” OR “coaching” OR “flexibility” OR “self confidence” OR “integrity” OR “vision” OR “enterpreneur* OR “conversation” OR “nonverbal communication” OR “body language” OR “presence” OR “mentor*” OR “personality development” OR “self concept” OR personal boundaries” OR “identity crisis” OR individuation” OR “intelligence” OR “reflect*“ OR “evidence based practice” OR “evidence based nursing”)	
	**#1 AND #2 AND #3**	**1.862**
	**Filter by publication date <10 year**	**1.098**
	**PsyINFO**	
#1 Target group	evidence based practice/ OR (BSN OR MANP OR advanced nursing practice).ti,ab. OR (((advanced practice OR Baccalaureate OR Bachelor OR Bachelor’s OR Master* OR practitioner* OR science OR scientist* OR specialist* OR Undergraduate).ti,ab.) AND (nurses/ OR nursing education/ OR nursing/ OR (nurse OR Nurses OR Nursing).ti,ab.))	**34.418**
#2 Work context	exp Nursing Homes/ or residential care institutions/ or long term care/ or elder care/ OR (Elderly care OR Geriatric nurs* OR Gerontological nurs* OR Housing for the elderly OR Long term care OR Long term health care OR Long term healthcare OR Nursing home* OR Residential care OR Residential facilit* OR Retirement home*).ti,ab.	**35.291**
#3 Competencies	exp Professional Competence/ OR exp Performance/ OR exp Attitudes/ OR health personnel attitudes/ OR attitudes/ OR exp Cultural Sensitivity/ OR culture change/ OR exp employee attitudes/ OR exp job satisfaction/ OR social behavior/ OR leadership/ OR leadership style/ OR organizational behavior/ OR responsibility/ OR social acceptance/ OR social networks/ OR social perception/ OR social skills/ OR exp Health Knowledge/ OR professional development/ OR development/ OR exp Social Skills/ OR working conditions/ OR skill learning/ OR learning/ OR learning strategies/ OR personality development/ OR exp problem based learning/ OR exp decison making/ OR problem solving/ OR declarative knowledge/ OR inductive deductive reasoning/ OR reasoning/ OR exp “knowledge (general)”/ OR cognitive processes/ OR metacognition/ OR exp Intuition/ OR communication skills/ OR exp ability/ OR exp conversation/ OR communication/ OR nonverbal communication/ OR persuasive communication/ OR verbal communication/ OR mentor/ or identity crisis/ OR professional standards/ OR intelligence/ OR emotional intelligence/ OR thinking/ OR critical thinking/ OR logical thinking/ OR rationality/ OR reasoning/ OR personality/ OR self-actualization/ OR self-concept/ OR	**2.791.172**

	self-determination/ OR self-expression/ OR “self-monitoring (personality)”/ OR cognitive style/ OR reflectiveness/ OR feedback/<br>OR<br>(Competencies OR competenc* OR clinical competenc* OR nurse competenc* OR professional competence OR abilit* OR Expertise OR Capability OR skill* OR skills mix* OR skill mix* OR skillmix* OR Tasks OR role* OR Knowledge OR professional attitude OR Behaviour OR Behavior OR communicati* OR Leadership OR organizational OR organisational OR analytic* OR critical thinking OR awareness OR creativity OR empathy OR networking OR persuasiveness OR decisiveness OR coaching OR flexibility OR self confidence OR integrity OR vision OR enterpreneur* OR conversation OR nonverbal communication OR body language OR presence OR mentor* OR personality development OR self concept OR personal boundaries OR identity crisis OR individuation OR intelligence OR reflect* OR evidence based practice OR evidence based nursing). ti,ab.	
	**#1 AND #2 AND #3**	**799**
	**Filter by publication date <10 year**	**473**
	**Total number of articles [PubMed, CINAHL and PsyINFO]**	**2.646**
	**Removing duplicates via Endnote**	**1978**
